# Mid-day siesta in natural populations of *D. melanogaster* from Africa exhibits an altitudinal cline and is regulated by splicing of a thermosensitive intron in the *period* clock gene

**DOI:** 10.1186/s12862-017-0880-8

**Published:** 2017-01-23

**Authors:** Weihuan Cao, Isaac Edery

**Affiliations:** 1 Rutgers University, Center for Advanced Biotechnology and Medicine, Piscataway, NJ 08854 USA; 2 Department of Molecular Biology and Biochemistry, Rutgers University, Center for Advanced Biotechnology and Medicine, 679 Hoes Lane, Piscataway, NJ 08854 USA

**Keywords:** *Drosophila*, Sleep, Circadian, Temperature, Mid-day siesta, Splicing, Altitude, Thermal adaptation, dmpi8 intron, *Period* gene

## Abstract

**Background:**

Many diurnal animals exhibit a mid-day ‘siesta’, generally thought to be an adaptive response aimed at minimizing exposure to heat on warm days, suggesting that in regions with cooler climates mid-day siestas might be a less prominent feature of animal behavior. *Drosophila melanogaster* exhibits thermal plasticity in its mid-day siesta that is partly governed by the thermosensitive splicing of the 3’-terminal intron (termed dmpi8) from the key circadian clock gene *period* (*per*). For example, decreases in temperature lead to progressively more efficient splicing, which increasingly favors activity over sleep during the mid-day. In this study we sought to determine if the adaptation of *D. melanogaster* from its ancestral range in the lowlands of tropical Africa to the cooler temperatures found at high altitudes involved changes in mid-day sleep behavior and/or dmpi8 splicing efficiency.

**Results:**

Using natural populations of *Drosophila melanogaste*r from different altitudes in tropical Africa we show that flies from high elevations have a reduced mid-day siesta and less consolidated sleep. We identified a single nucleotide polymorphism (SNP) in the *per* 3’ untranslated region that has strong effects on dmpi8 splicing and mid-day sleep levels in both low and high altitude flies. Intriguingly, high altitude flies with a particular variant of this SNP exhibit increased dmpi8 splicing efficiency compared to their low altitude counterparts, consistent with reduced mid-day siesta. Thus, a boost in dmpi8 splicing efficiency appears to have played a prominent but not universal role in how African flies adapted to the cooler temperatures at high altitude.

**Conclusions:**

Our findings point towards mid-day sleep behavior as a key evolutionary target in the thermal adaptation of animals, and provide a genetic framework for investigating daytime sleep in diurnal animals which appears to be driven by mechanisms distinct from those underlying nighttime sleep.

**Electronic supplementary material:**

The online version of this article (doi:10.1186/s12862-017-0880-8) contains supplementary material, which is available to authorized users.

## Background

Animals exhibit daily cycles of wake and sleep that are partly governed by cell-based circadian (≅24 h) clocks or pacemakers [1, 2]. The circadian system is thought to mainly regulate the daily timing of wake and sleep episodes, whereas sleep behavior, such as duration and quality, is driven by a balance between sleep-promoting pathways and arousal circuits [3]. For diurnal animals, sleep is mainly segregated to the night and light generally acts as an acute arousal signal. However, many diurnal animals exhibit a mid-day siesta that is more prominent at higher temperatures, almost certainly a critical adaptive response to avoid the detrimental effects of unnecessary exposure to heat [4–6]. While much work has focused on using model systems to understand the cellular and molecular bases underlying nocturnal sleep [7], little is known about the mechanisms governing mid-day siesta and its role in the thermal adaption of animal behavior.

*D. melanogaster* is an excellent model system to study mechanisms governing circadian rhythms and sleep [8–10], and in addition undergoes thermal adjustments in its daily behavior [4, 11–15]. Like many animals, *Drosophila melanogaster* exhibits a bimodal activity pattern with major bouts in the morning and evening that are separated by a mid-day siesta, whereas the majority of sleep occurs at night [16]. As ambient temperature increases, *D. melanogaster* manifest a longer and more robust mid-day siesta, delayed and more nocturnal evening activity bout, and although less dramatic an earlier offset in the morning bout of activity [4]. We showed that this behavioral plasticity is partly governed by the thermal sensitive splicing of the 3’-terminal intron found in the key circadian clock gene termed *period (per)* [4, 17, 18]. Splicing of this intron (called dmpi8; *D. melanogaster per* intron 8) is progressively more efficient as daily temperatures decrease, leading to an increase in *per* mRNA levels [4]. The molecular underpinnings for this thermosensitivity in splicing is due to the dmpi8 intron having weak 5’ and 3’ splice sites (ss), suggesting that at higher temperatures binding of the spliceosome is reduced leading to a reduction in splicing efficiency [18]. Although it is not clear how alterations in the splicing efficiency of dmpi8 and the associated changes in *per* mRNA and protein levels lead to changes in daily activity patterns, recent evidence indicates that this splicing event operates in a clock-independent manner that modulates the balance between daytime sleep-promoting and wake-promoting pathways [19]. For example, on cool days the efficient splicing of dmpi8 leads to a decrease in the threshold for sensory-mediated arousal, which favors activity over sleep, thus enabling flies to remain active throughout the day. Conversely, the weak splicing of dmpi8 on warm days limits the influence of arousal circuits and in addition the combination of light and heat ‘directly’ suppresses activity and/or increases sleep propensity (termed ‘paradoxical masking’; [13, 19–21]), tipping the balance towards the sleep promoting pathways operating during the middle of the day. Thus, the mid-day siesta in *D. melanogaster* is very sensitive to being shaped by thermal cues.

*D. melanogaster* is thought to have originated in the lowlands of sub-Saharan Africa, possibly south central regions [22–24], where daytime temperatures are warm throughout the year, underscoring why it has a genetically programmed mid-day siesta. However, unlike many *Drosophila* species, *D. melanogaster* has successfully colonized a wide variety of temperate regions where local daytime temperatures exhibit more extreme fluctuations throughout the year and can be much cooler compared to lowland tropical climates [25]. The thermosensitive splicing of dmpi8 might have contributed to the wide-spread colonization of *D. melanogaster* by providing a mechanism that maximizes engaging in daytime behaviors when environmental conditions are favorable, such as cooler temperatures, while still maintaining the ability to minimize potential risks from heat [18]. A role for dmpi8 splicing in the natural variation of *D. melanogaster* wake-sleep profiles is also buttressed by the observation that several single nucleotide polymorphisms (SNPs) in the *per* 3’ untranslated region of wild-caught *D. melanogaster* populations from the east coast of the United States modulates dimpi8 splicing efficiency in a manner that is causally linked to variations in mid-day siesta [17].

A classic approach to study thermal adaptation is to use latitudinal and/or altitudinal clines to determine if the phenotypic trait shows geographical variation. Altitudinal gradients offer the advantage of a steep change in ambient temperature over a short geographical scale. Although not as well-studied as latitudinal clines, numerous phenotypic traits in animals, including *Drosophila,* exhibit altitudinal clines [26]. In this study we measured the wake-sleep patterns of *D. melanogaster* derived from natural populations captured at different altitudes from tropical regions of sub-Saharan Africa [23, 24, 27–29] (Additional file 1: Table S1). Over a wide range of temperatures *D. melanogaster* from high altitudes show a less robust mid-day siesta that is characterized by more fragmented sleep. The increased daytime sleep of low altitude flies is maintained in constant light conditions, indicative of a clock-independent mechanism. A previously identified SNP in the *per* 3’ UTR, characterized by two variant types [17], strongly affects mid-day sleep levels in both low and high altitude flies, reinforcing the notion that this splicing event plays a major role in the natural variation of daily wake-sleep patterns observed in the wild. Intriguingly, at cooler temperatures the less efficient splicing variant manifests elevated levels of dmpi8 intron removal in high altitude flies compared to low altitude flies suggesting that, at least for this group of flies, adaptation to the colder temperatures found at high altitudes involved mechanisms that selected for higher dmpi8 splicing efficiency, leading to reduced mid-day siesta. Finally, our findings establish *D. melanogaster* from different altitudes in Africa as attractive genetic populations to study the molecular basis for daytime sleep, which appears to be governed by mechanisms distinct from nocturnal sleep [30].

## Results

### *Drosophila* from high altitudes manifest more of a ‘cold’ phenotype in the daily distribution of activity

In total, we measured the daily wake-sleep cycles of dozens of individual flies for each of 91 independent *D. melanogaster* lines from 20 localities in 10 different countries from tropical Africa (Additional file 1: Table S1). The majority of the findings presented in this report are based on flies from Cameroon and Kenya because for both countries numerous independent lines from relatively low (range is 78-561 m; herein referred to as low altitude group) and high (range is 2169-2506 m; herein referred to as high altitude group) altitudes were available—prior studies have used even less extreme altitude ranges then used here for defining low and high altitude groups for *D. melanogaster* from sub-Saharan Africa; e.g., [31]. In addition, flies from Cameroon (west cost) and Kenya (east coast) differ significantly in longitude, and evidence based on genetic structure suggests that the ancestral range of *D. melanogaster* was eastern Africa followed by a more recent expansion west, indicating that flies from the west and east have significantly different life-histories [23, 24]. Observing similar altitudinal differences in a trait from spatially diverse groups of flies would suggest that the trait is critical in the adaptation of *D. melanogaster* to high altitudes. Although there is also a wide range in altitudes for the flies we obtained from Ethiopia (Table S1), we did not focus on them in this study as there was more variability in behavioral measures compared to the low and high altitude flies we analyzed from Cameroon and Kenya (data not shown). Nonetheless, Ethiopian flies still showed similar altitudinal differences in wake-sleep profiles as those observed for flies from Cameroon and Kenya (see below), and were included in our large scale behavioral analysis as a function of altitude (see Figs. 5 and 6a).

**Fig. 1:**
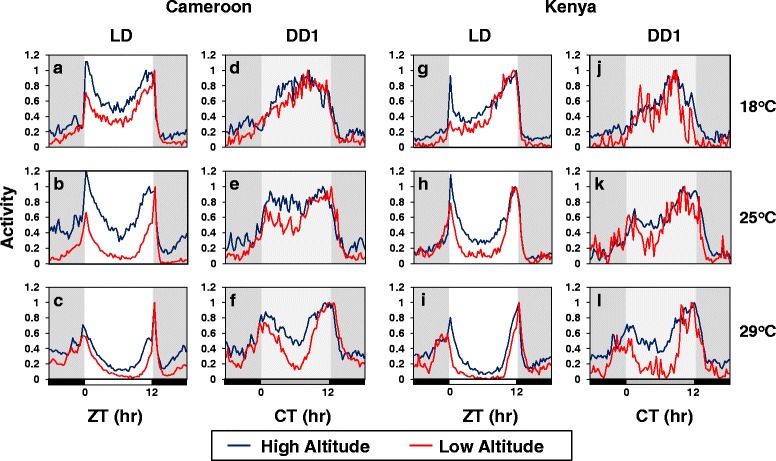
High altitude flies exhibit more of a ‘cold’ phenotype in daily wake-sleep profiles compared to low altitude flies. **a-l** Adult male flies from low and high altitudes representing several different locations in Cameroon (**a-f**) and Kenya (**g-l**) were kept at the indicated temperature (right of panels) and entrained to five days of 12 h light/12 h dark cycles (LD) followed by constant dark conditions (DD). For each country and temperature, the locomotor activity data of individual flies (16 flies per line) from the same altitude group (low or high) were pooled, and shown are group averages of daily activity rhythms (blue, high altitude group; red, low altitude group). To facilitate comparisons, the peak value in daily activity for each fly was set to 1.0 and the normalized profiles superimposed. For LD, the last three days’ worth of data was pooled; for DD, the first day is shown (DD1). The following fly lines were used in this experiment (see Additional file 1: Table S1 for further details); Cameroon low altitude (CM16, CM17, CM22, CM54, CY1); Cameroon high altitude (CO1, CO2, CO4, CO8, CO10, CO13, CO15, CO16); Kenya low altitude (KM10, KM16, and KM20); Kenya high altitude (KN5, KN6M, KN11M, KN13M, KN19M, KN23M, KO2, KO6, KO10M). Horizontal bars at bottom of panels denote 12-h periods of light (white bar), dark (black bar) and ‘subjective daytime’ in DD (gray bar). ZT, zeitgeber time (hr); CT, circadian time (hr). The results shown in this figure are representative of at least two independent experiments, using the same or additional lines from Cameroon or Kenya (see Additional file 1: Table S1). The results show that during LD high altitude flies from both Cameroon and Kenya exhibit a more pronounced ‘cold-type’ daily activity pattern, highlighted by a shorter and less robust midday siesta, earlier onset of evening activity and later offset of morning activity

**Fig. 2:**
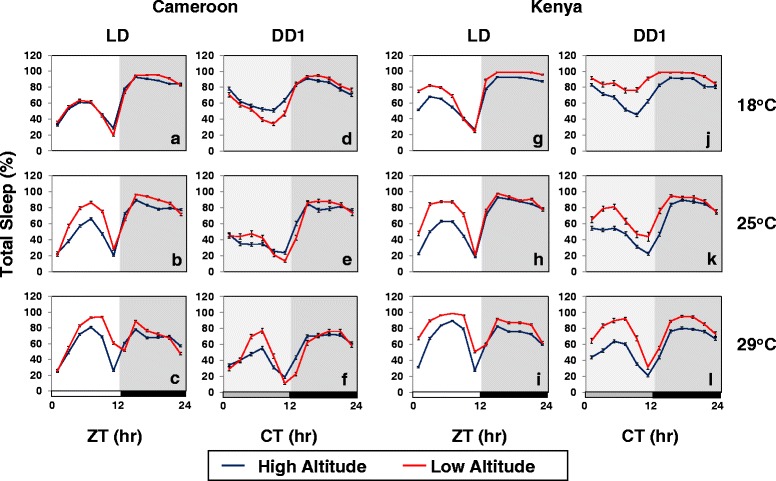
High altitude flies sleep less during the day. **a-l** The results are based on the same flies and activity data used in Fig. 1 and show group averages for daily sleep levels (expressed as the percentage of time flies are sleeping during 2 h time-windows) for either high altitude (*blue line*) or low altitude (*red line*) adult male flies from Cameroon and Kenya maintained at the indicated temperatures (*right* of panels) and light/dark conditions (*top* of panels). For LD, sleep data from the last 3 days were pooled, whereas for DD, the first day is shown (DD1). Horizontal bars at bottom of panels denote 12-h periods of light (white bar), dark (black bar) and ‘subjective daytime’ in DD (gray bar). ZT, zeitgeber time (hr); CT, circadian time (hr)

To analyze daily wake-sleep profiles, flies were exposed to at least 5 days of the standard entraining conditions of 12 h light followed by 12 h dark [LD; where zeitgeber time 0 (ZT0) is lights-on], followed by 7 days of total darkness (DD) to measure free-running rhythms [16, 32]. In general, daily wake-sleep cycles were measured at the standard temperature of 25 °C, in addition to 18° and 29 °C based on prior studies showing that mid-day siesta is less pronounced on colder (e.g., 18 °C) compared to warmer (e.g., 29 °C) days [4, 19]. Finally, behavioral studies were mainly done with male flies as they usually generate more robust wake-sleep rhythms with the monitoring system used [16, 32]. Importantly however, although mid-day siesta levels are typically lower in females [10, 33], the results we obtained were very similar in males and females (e.g., Figs. 1, 2 and Additional file 2: Figure S1).

We first analyzed the daily distribution of locomotor activity, and show results obtained with the low and high altitude groups from Cameroon and Kenya (Fig. 1). Consistent with prior work, *D. melanogaster* flies from equatorial Africa exhibit a bimodal distribution of activity at the standard temperature of 25 °C in LD, with prominent morning (around ZT0) and evening (around ZT12) bouts that are separated by a mid-day dip in activity or siesta (Fig. 1b and h) [34]. Moreover, all flies show the standard *D. melanogaster* daytime response to increases in daily temperature, characterized by a longer and more robust mid-day siesta, in addition to delayed upswing in the evening bout of activity (Fig. 1; a-c and g-i; data not shown) [4]. Thus, the ability to respond to temperature by modulating the daily distribution of activity appears to be a universally conserved response in natural populations of *D. melanogaster*. However, high altitude flies manifested daily activity profiles that are relatively more characteristic of a ‘cold’ phenotype compared to their low altitude counterparts (Fig. 1; a-c and g-i). Specifically, at all temperatures tested high altitude flies showed a less robust and shorter mid-day siesta, advanced onset of evening activity and delayed offset of morning activity compared to their low altitude counterparts.

While flies from Cameroon and Kenya exhibit remarkably common trends in daily activity patterns with changes in temperature and altitude, there are some aspects that are not as well conserved. For example, although there were little to no altitudinal differences in nighttime activity profiles (i.e., ZT12-24/0) for flies from Kenya (Fig. 1. g-i, grey region of activity profiles), nocturnal activity levels were typically relatively higher in high altitude flies from Cameroon compared to the low altitude group (Fig. 1, a-c).

To determine if altitudinal differences in daytime activity profiles are modulated by light we also analyzed behavioral rhythms during the first day in total darkness (DD1) following entrainment to LD (Fig. 1, compare LD to DD1). At the lower temperatures of 18° and 25 °C, the daily activity patterns of low and high altitude flies were more similar during DD1 compared to their corresponding LD profiles (Fig. 1, e.g., compare panels b and e). Thus, exposure to light enhances the altitudinal differences in daytime activity profiles, especially at cooler temperatures. It should be noted that although light can act as an acute arousal cue, over the course of a daily cycle it also acts to suppress activity in *Drosophila*, especially in the mid-day and in combination with warm temperatures [13, 19, 21, 35, 36]. The ability of light and warm temperature to suppress activity has been observed in other animals besides *Drosophila* and is termed ‘paradoxical masking’ [37]. This inhibitory effect of daytime light on activity levels is clearly observed in African flies as there is a stronger mid-day dip in activity during LD compared to DD1 (Fig. 1). In this vein it is interesting to note that at 29 °C the mid-day siesta in low altitude flies is significantly more prominent compared to high altitude flies even in the absence of daily light (Fig. 1, panels f and l). Thus, irrespective of the inhibitory effects of light on overall daytime activity levels, flies from low altitudes appear to be preferentially ‘hard-wired’ to suppress mid-day activity on warm days compared to high altitude flies.

In DD1 both low and high altitude flies exhibit delayed timing in the peak of evening activity, consistent with prior work [4]. This further supports the observations in LD that both low and high altitude flies from Africa exhibit similar trends in daily wake-sleep patterns as a function of temperature but mainly differ in mid-day behavior. By measuring daily behavioral cycles in DD we also observed that the period length of these rhythms are approximately 30 min longer in the high altitude flies from Cameroon and Kenya (Table S2). Longer free-running periods are normally associated with delayed timing in the evening peak (e.g., [38]), further emphasizing that the shorter mid-day siesta and advanced evening activity bout of high altitude flies are not due to differences in circadian properties (see below).

### Daytime sleep in high altitude flies is shorter and more fragmented compared to low altitude flies

Sleep in *Drosophila* is characterized by multiple bouts of extended quiescence that can range in length and number [33]. We used the standard operational definition of sleep as 5 contiguous min of no recorded locomotor movement [19, 33, 39, 40]. Low altitude flies exhibit significantly higher daytime sleep levels, especially in the mid-day, compared to their high altitude counterparts (Fig. 2, a-c, g-i; and data not shown). This trend was generally observed over a wide range of temperatures (18° to 29 °C), except for Cameroon flies at 18 °C (Fig. 2a; however, see below Fig. 7). Nighttime sleep levels were generally similar or slightly increased in low altitude flies, indicating that altitude has a relatively larger effect on daytime sleep levels (see also Fig. 5). As with activity profiles, some differences in the daily sleep patterns of flies from Kenya and Cameroon were observed. Most notably, the altitudinal differences in daily sleep observed during LD persisted in the absence of light for flies from Kenya, whereas the daily sleep levels of low and high altitude flies from Cameroon were more similar in DD1 (Fig. 2, d-f and j-l). Female flies from Cameroon and Kenya showed the same altitudinal differences in daily sleep profiles in LD and DD1 as observed for males (Additional file 2: Figure S1). Thus, it appears that a widely conserved adaptation of high altitude flies in tropical Africa is reduced daytime sleep levels.

**Fig. 3:**
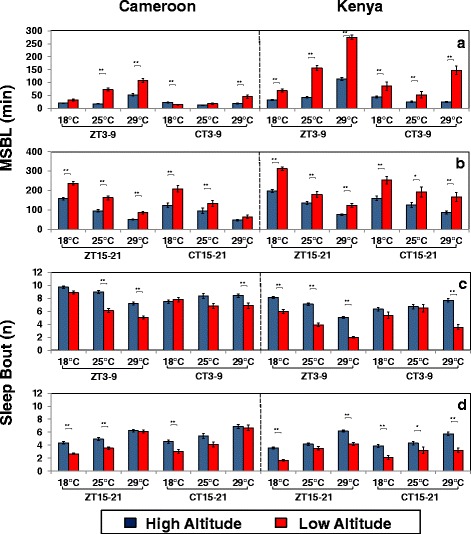
Daytime and nighttime sleep are more fragmented in high altitude flies. **a-d** Shown are group averages for median sleep bout length (MSBL) (**a**, **b**) and number of sleep bouts (**c**, **d**) for either high (*blue bar*) or low (*red bar*) altitude flies from Cameroon and Kenya at the indicated temperatures and 6-h time interval during LD (i.e., ZT3-9 or ZT15-21) or DD1 (i.e., CT3-9 or CT15-21) (*bottom of panels*). The results shown are based on the same flies and activity data used to generate Figs. 1 and 2. ZT3-9 and ZT15-21 denote 6-h periods during the mid-day and mid-night in LD, respectively; CT3-9, and CT15-21 denote 6-h periods of ‘subjective mid-day’ and ‘subjective mid-night’ in DD1, respectively. Values for high altitude and low altitude flies are significantly different using student’s *t-test*; *, *p* < 0.05; **, *p* < 0.01

Reductions in total sleep levels can be due to decreases in the average length of sleep episodes and/or the number of sleep bouts [33]. To more specifically compare the effects of altitude on mid-day sleep behavior we focused on a 6 h time-window spanning either the middle of the day during LD (i.e., ZT3-9) or the ‘subjective’ day during DD1 [i.e., CT3-9 (CT; circadian time in DD)] (Fig. 3a and c). In addition, we also measured nighttime sleep behavior during an ‘equivalent’ 6 h time-window during the mid-night (i.e., ZT/CT15-21) (Fig. 3b and d).

Over a wide range of temperatures, high altitude flies exhibit significantly shorter sleep bout lengths during the mid-day in LD compared to low altitude flies (Fig. 3a; see values for ZT3-9). In addition, although not as prominent as the altitudinal effect on average sleep bout length, high altitude flies have more sleep bouts during the mid-day in LD (Fig. 3c; see values for ZT3-9). Interestingly, prior work showed that the main effect of heat on sleep behavior is on the length of sleep episodes, with sleep bout number less affected [19, 20]. The combination of an increased number of shorter sleep bouts indicates that mid-day sleep in high altitude flies is less consolidated compared to low altitude flies (e.g., [41]). The only outlier we noticed in this trend were Cameroon flies at 18 °C, similar to the lack of altitudinal differences in total daytime sleep levels (Fig, 2a; however, see Fig. 7). Although at any given temperature sleep bout length during the mid-day in LD is shorter in high altitude flies compared to their low altitude counterparts, both groups exhibited longer sleep bouts as temperatures rise (Fig. 3a), consistent with prior reports that mid-day sleep is enhanced at warmer temperatures [19, 30]. In agreement with total sleep levels (Fig. 2), altitudinal differences in the average length of a mid-day sleep bout during the first day of constant dark conditions were more prominent for flies from Kenya compared to Cameroon (Fig. 3a; compare values for ZT3-9 to CT3-9). Finally, sleep bout length during the mid-day is generally longer in the presence of light, indicative of the direct inhibitory or ‘paradoxical masking’ effect of daytime light on activity (Fig. 3a; compare ZT3-9 and CT3-9 values), as previously noted [13, 19, 20].

Although total nighttime sleep levels showed little to no altitudinal variation (Fig. 2), the average sleep bout was shorter and there were more sleep bouts in high altitude flies, a trend that continued in DD1 (Fig. 3b and d, compare values for ZT15-21 and CT15-21). Thus, even though altitudinal differences in total sleep levels are relatively larger during the day (Fig. 2), both daytime and nighttime sleep are less consolidated in high altitude flies compared to low altitude flies (see Discussion). However, whereas the average sleep bout during the mid-day progressively lengthens with increases in temperature, the opposite trend was observed for nighttime sleep (Fig. 3a and b; compare values for ZT3-9 and ZT15-21). This reciprocal relationship of the effects of temperature on daytime and nighttime sleep has been noted before (e.g., [20, 30]). It is possible that the enhanced sleep during the mid-day at warmer temperatures reduces the intensity of nighttime sleep [20]. Altitude had little effect on how active flies were during wake periods (data not shown), suggesting that the the altitudinal differences in sleep behavior we observed are not due to health issues or hyper-activity.

Intriguingly, the altitudinal differences in daily wake-sleep profiles are reminiscent of those we obtained when we analyzed transgenic flies that carry either a wildtype version of the dmpi8 intron (termed 8:8) versus those with a modified version that splices much more efficiently (termed M2M1) [19]. Similar to how high altitude flies differ from low altitude flies in daily wake-sleep profiles, the M2M1 flies exhibit more of a cold phenotype compared to wildtype transgenics, including an advanced evening activity bout and reduced mid-day sleep that is more fragmented. In addition, the major effect of high altitude on mid-day sleep behavior is on shortening the average length of a sleep episode, with relatively less effect on sleep bout number (Fig. 3). This is similar to what we previously observed with the M2M1 flies compared to the wildtype transgneics [19], suggesting that like the M2M1 flies, daytime sleep in high altitude flies is less intense.

### Sleep differences between low and high altitude flies continue in constant light conditions

**Fig. 4:**
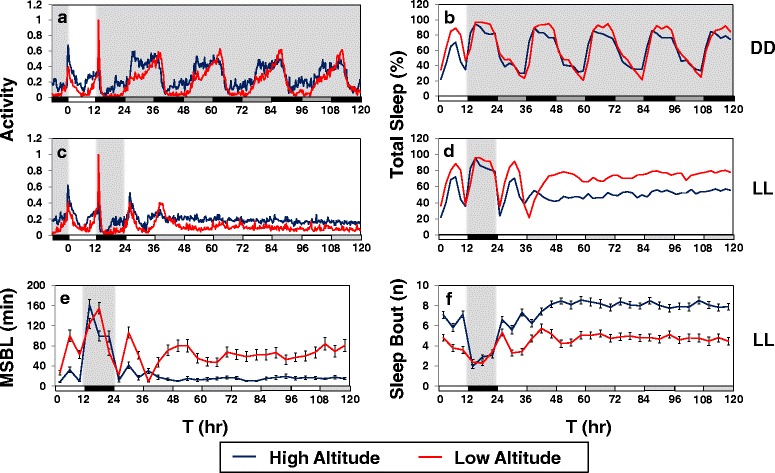
Reduced and more fragmented sleep in high altitude flies continues even in constant light conditions where circadian clock function is abolished. **a-f** Shown are group averages of fly activity (**a** and **c**), sleep levels (**b** and **d**), median sleep bout length (**e**), and number of sleep bouts (**f**) for Cameroon male flies from either the high (*blue line*) or low (*red line*) altitude groups. For this experiment, two representative lines were used for each altitude; Cameroon low altitude (CO1, CO4); Cameroon high altitude (CM16, CM54). Flies were maintained at 25 °C and entrained for five days in LD. Subsequently, half of the high and low altitude groups were placed in constant darkness (DD), whereas the other half was placed in constant light (LL). White, black, dark gray, and light gray horizontal bars below panels represent 12-h periods of light, dark, ‘subjective daytime’ in DD, and ‘subjective nighttime’ in LL, respectively. In LL, comparison of low and high altitude flies for daily activity profiles (**c**), daily sleep levels (**d**), median sleep bout length (**e**), and number of sleep bouts (**f**) showed highly significant differences (one-way ANOVA, *p* < 0.0001). Similar results showing that high altitude flies exhibit less total sleep that is more fragmented in LL compared to low altitude flies were also obtained with flies from Kenya (data not shown)

In prior work we showed that the decreased daytime sleep of M2M1 flies compared to wildtype transgenic controls persists in extended constant light (LL) [19], conditions which conditionally abrogate clock function and circadian rhythms (e.g., Fig. 4, compare panels a and c; activity rhythms are abolished after the first day of LL but persist in DD) [42]. Likewise, in extended LL sleep rhythms are abolished (Fig. 4, compare b and d), and high altitude flies from either Cameroon or Kenya have significantly lower total sleep levels compared to low altitude flies (Fig. 4d, and data not shown). Moreover, not only is the average length of a sleep bout very short in high altitude flies during LL but there is also an increase in the total number of sleep bouts (Fig. 4e and f), indicating that high altitude flies have a severe deficit in maintaining sleep during constant light conditions. Thus, the more fragmented sleep of high altitude flies in the presence of light does not require a functional clock.

In summary, while not all the altitudinal differences in sleep behavior during LD, DD or LL are shared between flies from Cameroon and Kenya (or at all temperatures) there are some noteworthy similarities. Most notably, the mid-day sleep of high altitude flies in daily light–dark cycles is shorter and more fragmented, a difference in sleep behavior that continues in constant light indicative of a clock-independent mechanism. With regards to nighttime sleep, although total levels are not as affected by altitude, similar to daytime sleep it is more fragmented in flies from high altitudes.

### Altitudinal cline in sleep behavior is widespread in natural populations of flies from tropical Africa

**Fig. 5:**
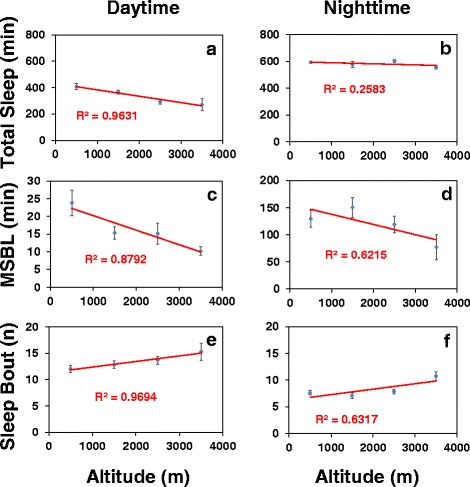
Altitudinal cline in daytime sleep behavior is widely observed in flies from tropical Africa. **a-f** Shown are group averages of total sleep (**a**-**b**), median sleep bout length (**c**-**d**), and number of sleep bouts (**e-f**) during the 12-h period of either daytime or nighttime in LD for a total of 91 independent lines (16 flies per line) from 20 localities in 10 different countries (see Additional file 1: Table S1). Flies were exposed to at least 5 days of LD at 25 °C and activity data from the last three days of LD was pooled and analyzed in 1000 m increments. The lines in the panels represent the regression analysis of phenotypic means (y-axis) as a function of altitude (x-axis). The results show that there is a more pronounced altitudinal cline in daytime sleep levels and quality compared to nighttime sleep, although the daytime trend of more fragmented daytime sleep with increasing altitude is still observed with nighttime sleep

In more limited experiments, we also measured the daily wake-sleep cycles of flies from tropical regions of Africa besides Kenya and Cameroon (Additional file 1: Table S1). A total of 91 independent lines from 20 localities in 10 different countries were entrained under the standard conditions of 12 h light followed by 12 h dark at the standard temperature of 25 °C for at least 5 days and daily wake-sleep cycles measured. Because altitudes vary greatly in the populations analyzed (78-3070 m), but for some altitudes we only had one or a few independent lines (Additional file 1: Table S1), we pooled the results in bins of 1000 m to simplify the analysis (Fig. 5). Although pooling data in large increments affects the significance of the overall regression analysis as a function of altitude, the results show similar altitudinal trends as those observed with flies from Cameroon and Kenya. Most notably, i) altitude preferentially modulates total daytime sleep levels with little to no effect on total nighttime sleep (Fig. 5, a and b); ii) as altitude increases the average sleep bout length during the day becomes progressively shorter (panel c) and to a lesser extent there are more of them (panel e); and iii) although total nighttime sleep levels are less influenced by altitude, nighttime sleep is progressively more fragmented (i.e., increased number of shorter sleep bouts) as altitude increases (panels d and f).

### SNP3 in the 3’ UTR of *per* has strong effects on the mid-day sleep behavior and dmpi8 splicing efficiency of African flies, especially those from low altitudes

In prior work we identified four SNPs (termed SNPs 1–4) in the *per* 3’ UTR from natural populations of flies located in the eastern coast of the United States, some of which we showed affect dmpi8 splicing efficiency and mid-day siesta [17]. Based on our own sequencing analysis of *per* 3’ UTRs and/or published databases we obtained *per* 3’ UTR sequence information from 67 of the 91 lines we analyzed behaviorally (data not shown and Additional file 1: Table S1). We identified at least 5 novel SNPs in flies from tropical Africa in addition to the four we previously reported for flies from the eastern coast of the United States (data not shown). Unfortunately, preliminary analysis did not reveal any clear altitudinal variations in the frequency of any individual or combinations of SNPs in the *per* 3’ UTRs of African flies (data not shown). However, a more definitive conclusion about SNP frequency and function will require expanding the number of localities and determining the effects of different SNP combinations on dmpi8 splicing and wake-sleep profiles, areas of investigation that are ongoing.

**Fig. 6:**
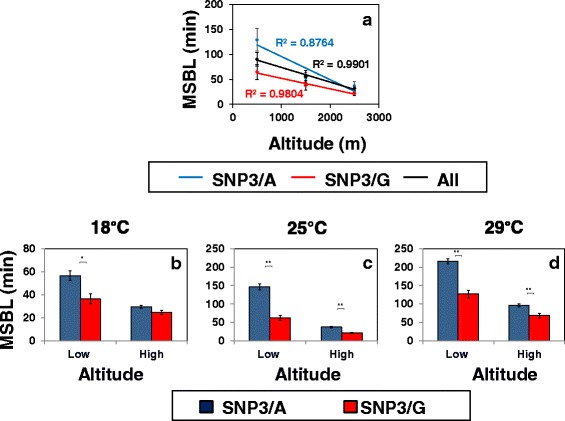
SNP3/A flies exhibit a larger altitudinal effect on mid-day sleep compared to the SNP3/G variant. **a** Shown are the group averages of median sleep bout length during the mid-day (ZT3-9) for flies with SNP3/A (*blue dot* and *line*), SNP3/G (*red dot* and *line*), or the combination of all 91 lines tested (*black dot* and *line*), as a function of altitude in 1000 m increments. The results shown are based on the same activity data used to generate Fig. 5, except that for SNP3/A and SNP3/G flies we only used data from the 67 independent lines where the identity of the SNP3 variant was known (see Additional file 1: Table S1). Briefly, flies were exposed to at least 5 days of LD at 25 °C and activity data from the last three days of LD was pooled. The lines in the panel represent the regression analysis of phenotypic means (y-axis) as a function of altitude (x-axis). **b**-**d** Shown are group averages of median sleep bout length during the mid-day (ZT3-9) for flies from Cameroon and Kenya that were exposed to at least 5 days of LD at the indicated temperature (*top* of panels). The results are based on the same flies used in Fig. 1; listed according to altitude group and SNP variant the flies analyzed were; low altitude SNP3/A (CM16, KM10, KM16, KM20); high altitude SNP3/A (CO4, CO8, CO16, KN5, KN6M, KN11M, KN19M, KO2, KO6, KO10M); low altitude SNP3/G (CM17, CM22, CM54, CY1); high altitude SNP3/G (CO1, CO2, CO10, CO13, CO15, KN13M, KN23M). Values for SNP3/A and SNP3/G flies are significantly different using student’s *t-test*; *, *p* < 0.05; **, *p* < 0.01

**Fig. 7:**
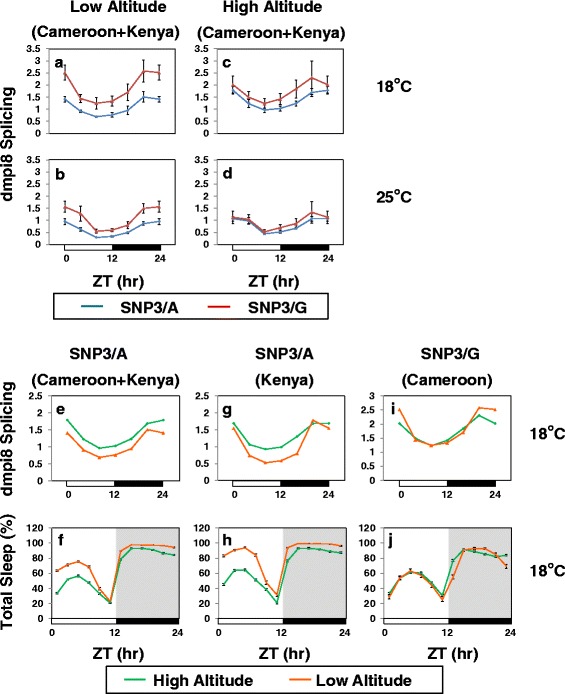
Strong association between daily levels of dmpi8 splicing and mid-day sleep at cold temperatures. **a-d** Shown are group averages for the splicing efficiency of dmpi8 (expressed as the ratio of spliced to unspliced levels) throughout an LD cycle for low and high altitude flies from Cameroon and Kenya pooled according to the SNP3 variant. The same fly lines as those used for the sleep analysis shown in Fig. 6b and c (see legend to Fig. 6) were also used to measure dmpi8 splicing, allowing for comparison between the behavioral and molecular results. Briefly, for each isofemale line approximately 40 flies were placed into each of 12 vials. Half of the vials were exposed to 5 days of LD at 18 °C, whereas the other half was exposed to 5 days of LD at 25 °C. On the last day of LD, flies were collected by removing a vial every 4 h, total RNA prepared and dmpi8 splicing efficiency measured for each line separately, followed by pooling results from different lines to yield group averages. For low altitude flies, the daily dmpi8 splicing curves at 18° and 25 °C were significantly different between the SNP3/A and SNP3/G variants (18 °C; one-way ANOVA, *p* < 0.01; 25 °C, one-way ANOVA, *p* < 0.05), whereas no significant differences were observed for the dmpi8 splicing curves from high altitude flies. **e**, **g**, **i** Using the same splicing data shown above for flies at 18 °C (**a**, **c**), the data from individual fly lines was sorted to compare the dmpi8 splicing curves from the low and high altitude groups with the same SNP3 variant. The error bars were removed to facilitate comparison. Comparison of the daily dmpi8 splicing curves for low and high altitude flies with SNP3/A (**e**, **g**) were significantly different (one-way ANOVA, *p* < 0.01) but no significant difference was observed for the comparison using SNP3/G flies from Cameroon (**j**). Directly below each splicing panel (**e**, **g**, **i**) is shown the corresponding daily sleep profile (**f**, **h**, **j**) for the same group of flies used to generate the splicing results. Similar results were obtained in several smaller scale experiments (data not shown)

Nonetheless, SNP3 was identified as having prominent effects on dmpi8 splicing and mid-day siesta in flies from the eastern coast of the United States ([17]; and data not shown). SNP3 has two variants, either a G or an A residue, and we showed that the SNP3/G variant is causally linked to more efficient dmpi8 splicing, consistent with reduced mid-day siesta ([17]; see also Fig. 7; and data not shown). Of the African lines that we had *per* 3’ UTR sequence information, about 40% carry the SNP3/G variant and the rest are SNP3/A (Additional file 1: Table S1). We wondered if SNP3 also has prominent effects on daytime sleep behavior in natural populations of African flies, which includes more ancestral and genetically diverse strains compared to flies from the more recently colonized United States (e.g., [43]). Indeed, at lower altitudes the duration of mid-day sleep bouts is much shorter for flies with SNP3/G compared to SNP3/A, and as expected the values for all the 91 strains analyzed fell in between the values for SNP3/A and SNP3/G flies (Fig. 6a). However, at the highest altitudes analyzed both the SNP3/A and SNP3/G flies have very short sleep bouts, suggesting this SNP is not a major contributor to the observed altitudinal differences in mid-day siesta, consistent with the lack of an altitudinal difference in the relative frequency of SNP3/A and SNP3/G (Additional file 1: Table S1 and data not shown). Thus, it appears that irrespective of any other genetic diversity between strains, SNP3/G has dominant effects in lowland flies resulting in strongly reduced mid-day sleep compared to those with the A variant. The already reduced mid-day sleep in SNP3/G flies even at low altitudes likely acts as a ‘ceiling’ limiting how much further mid-day sleep can be decreased at higher altitudes (see below).

The effects of the SNP3 variant on mid-day sleep and its larger influence in low altitude flies was also observed when we limited our analysis to include only flies from the better characterized Cameroon and Kenya populations (Fig. 6c). More extensive analysis of the Cameroon and Kenya flies further showed that the reduced mid-day sleep bout length of flies carrying the SNP3/G variant compared to the SNP3/A variant was also observed at 18° (Fig. 6b) and 29 °C (Fig. 6d). In addition, the difference in sleep bout length between SNP3/A and SNP3/G flies was greater for low altitude flies at 18° and 29 °C, similar to that observed at 25 °C.

Based on these behavioral results we measured dmpi8 splicing efficiency in flies from the low and high groups from Cameroon and Kenya entrained to 18° and 25 °C (Fig. 7a-d). There are several noteworthy aspects of the dmpi8 splicing results that are strikingly consistent with the behavioral results, especially at the lower temperature. For example, the daily dmpi8 splicing efficiency is higher for SNP3/G flies, consistent with their reduced mid-day sleep (Fig. 6). Moreover, with regards to altitude, the difference in the daily levels of dmpi8 splicing between SNP3/G and SNP3/A flies was greater in low altitude flies compared to high altitude flies (Fig. 7, compare panels a and c; and b to d), similar to the effects on mid-day sleep (Fig. 6b and c). For both SNP3/A and SNP3/G flies, the daily splicing efficiency of dmpi8 is higher at 18 °C compared to 25 °C (Fig. 7a-d), as expected based on prior work showing that increases in temperature progressively inhibit dmpi8 splicing [4, 14, 18, 44, 45]. Also in agreement with prior work, the daily curve in dmpi8 splicing efficiency exhibits a low amplitude cycle with trough values reached during the mid-day [44, 45].

That the difference in dmpi8 splicing levels for SNP3/A and SNP3/G variants is better observed at 18 °C compared to higher temperatures (Fig. 7; compare a to b, and c to d) was previously noted using flies from the United States [17]. As discussed previously, this is not surprising based on our earlier findings showing that the strong inhibitory effects of warm temperatures on dmpi8 splicing efficiency are due to weak 5’ and 3’ss which are identical in all natural *D. melanogaster* strains ([18]; and data not shown). Therefore, at higher temperatures the suboptimal 5’ and 3’ss ensure that dmpi8 splicing is strongly inhibited, reducing the modulatory effects of SNPs or other factors on influencing the efficiency of intron removal. In addition, we previously showed that light in combination with heat can directly suppress mid-day activity (paradoxical masking) in a manner that largely overrides dmpi8 splicing [19]. Otherwise stated, although there is an altitudinal effect on daily wake-sleep profiles over a wide range of temperatures (i.e., 18° to 29 °C; Figs. 1 and 2), differences in the intrinsic splicing efficiency of dmpi8 almost certainly have relatively larger effects in shaping mid-day sleep behavior at cooler temperatures, whereas other factors such as direct masking effects of light and heat also contribute at higher temperatures [19, 20].

### Altitudinal differences in dmpi8 splicing efficiency suggest a role in adapting to the cooler temperatures at higher altitudes

Intriguingly, the splicing results comparing low and high altitude flies according to the SNP3 variant (Fig. 7a-d), also showed that at 18 °C the overall daily splicing efficiency of dmpi8 in SNP3/A flies is clearly elevated at high altitudes compared to low altitudes (Fig, 7e). Again, altitudinal differences in the daily splicing profiles were more apparent at 18 °C compared to 25 °C (data not shown), similar to what we observed with the comparison between SNP3/A and SNP3/G flies (Fig. 7a-d). This was further confirmed when we analyzed SNP3/A flies from only Kenya (Fig. 7g), which have multiple lines of low and high altitude flies (Additional file 1: Table S1). In contrast, the daily dmpi8 splicing curves for low and high altitude flies with the SNP3/G variant were very similar throughout most of a daily cycle (Fig. 7i), indicating that relative to SNP3/A flies, altitude has less effect on dmpi8 splicing efficiency in SNP3/G flies. Unfortunately, we could not do a similar analysis for SNP3/G flies from Kenya as none of the low altitude flies had this variant (Additional file 1: Table S1).

Nonetheless, similar to what we noted with the SNP3 variant at cooler temperatures, there is a remarkable link between the effects of altitude on the overall daily splicing efficiency of dmpi8 and mid-day sleep at 18 °C (Fig. 7, e-j). Indeed, the little to no effect of altitude on dmpi8 splicing in SNP3/G flies (Fig. 7i) combined with the high prevalence of SNP3/G in flies from Cameroon (Additional file 1: Table S1) fits nicely with the absence of an altitudinal effect on daytime sleep for flies from Cameroon at 18 °C (Fig. 2a and 7j). Conversely, for the low and high altitude flies from Kenya the far majority are SNP3/A (Additional file 1: Table S1), consistent with altitudinal effects on dmpi8 splicing (Fig. 7g) and daily sleep (Figs. 2g and 7h). Thus, although the effects of altitude on dmpi8 splicing are less dramatic than those between SNP3/A and SNP3/G (i.e., Fig, 7, compare a and e), at colder temperatures there is a strong correlation between the effects of altitude on dmpi8 splicing and daytime sleep levels. Based on our prior work showing a direct causal relationship between increased dmpi8 splicing efficiency and reduced mid-day sleep at cooler temperatures [18], our findings strongly suggest that the decreased daytime sleep of high altitude SNP3/A flies at cooler temperatures is at least partly due to increased levels of dmpi8 splicing compared to the low altitude group.

Thus, with regards to mid-day sleep there appears to be at least two major groups of flies from tropical Africa; those that have SNP3/G and those with SNP3/A. The influence of SNP3 on mid-day sleep levels appears to vary as a function of altitude and temperature. In lowland flies, SNP3/G has dominant effects on enhancing dmpi8 splicing efficiency and decreasing mid-day siesta resulting in more of a ‘cold’ phenotype in wake-sleep profiles compared to SNP3/A flies. This is similar to what we previously observed in flies from the eastern coast of the United States [17]. In flies adapted to high altitudes, SNP3/G flies still manifest higher dmpi8 splicing efficiency and reduced mid-day sleep levels compared to SNP3/A flies, but the differences are reduced. Altitudinal differences in mid-day sleep for SNP3/G flies, although less then those in SNP3/A flies, were still observed but do not appear to involve changes in dmpi8 splicing efficiency. In contrast, high altitude flies with the SNP3/A variant exhibit higher daily dmpi8 splicing efficiency compared to their lowland counterparts, especially at cooler temperatures.

Taken together, these observations suggest the following speculative model: Genetic changes that boost the efficiency of dmpi8 splicing beyond that observed in lowland flies can contribute to high altitude adaptation by reducing mid-day sleep levels. Increasing the strengths of the 5’ and 3’ss flanking the dmpi8 intron can augment splicing efficiency [18], however, all dmpi8 introns analyzed to date have the same suboptimal 5’ and 3’ss (data not shown). It is likely that increasing the 5’ and 3’ss to attain cold acclimation might be detrimental at warm temperatures as it would diminish the ability to mount a robust mid-day siesta [18]. Thus, the weak 5’ and 3’ss set a fixed upper limit to how much dmpi8 splicing efficiency can be increased in nature. In SNP3/A flies the lower baseline splicing efficiency of dmpi8 offers a genetic foundation wherein evolutionary forces can yield significant increases in dmpi8 splicing efficiency at high altitudes. Thus, the ability to generate significant altitudinal differences in dmpi8 splicing efficiency at cooler temperatures allowed this splicing event to play a prominent role in the high altitude acclimation of daytime behavior in SNP3/A flies. However, this evolutionary route was not available to SNP3/G flies because they already have high dmpi8 splicing efficiency even in lowland flies. Since the majority of flies from tropical Africa appear to have the SNP3/A variant, altitudinal differences in dmpi8 splicing efficiency likely played a major but not universal role in the adaptation of mid-day sleep behavior to high altitudes. Why dimpi8 splicing is more efficient in high altitude SNP3/A flies is presently not known. Clearly, other factors besides dmpi8 splicing contribute to the adaptation of mid-day sleep levels at high altitudes, especially for SNP3/G flies. Thus, although SNPs in the *per* 3’ UTR do not directly underlie altitudinal differences in daytime sleep behavior, they contribute to intrinsic differences in dmpi8 splicing splicing efficiency that as a result modulates the magnitude of altitudinal differences in mid-day siesta levels and likely influenced the evolutionary paths used to attain these differences.

## Discussion

This study was initiated based on the observation that many diurnal animals living in warm climates exhibit a mid-day siesta that exhibits thermal plasticity. Might the long-term adaptation to cooler climates for species that originated in warm climates involve a diminished mid-day siesta where the threat from excessive heat is minimized? In this regard *D. melanogaster* offers an attractive animal model to study the function and mechanism underlying mid-day sleep. Although it originated in the eastern lowlands of tropical Africa [22–24] where a robust genetically programmed mid-day sleep is advantageous, it has successfully colonized temperate regions with cooler temperatures. Using natural populations of *D. melanogaster* from tropical regions of Africa, we show that over a wide range of temperatures flies from high altitudes exhibit more of a ‘cold’ daily wake-sleep profile with a reduced mid-day siesta (Figs. 2 and 3), suggesting that mid-day siesta is a key target of natural selection underlying the ability of some animals to expand their geographical distribution to new temperature ranges.

The reduced total daytime sleep of flies from high altitudes is characterized by an increase in more frequent but shorter sleep bouts, indicative of less consolidated or intense sleep (Fig. 3). Although total nighttime sleep levels did not show an altitudinal cline (Figs. 2 and 5), it was also more fragmented in high altitude flies (Figs. 3 and 5), suggesting a general effect of decreased intensity for all sleep at higher altitudes. Currently, it is not clear if there is biological significance to the more fragmented nighttime sleep in high altitude flies as total sleep levels were not affected and was not the focus of this study. The more fragmented sleep in high altitude flies could arise from a reduction in the relative strength of sleep-promoting pathways and/or an increase in the relative influence of wake-promoting pathways. Future studies will be required to determine if arousal thresholds are altered as a function of altitude.

Importantly, high altitude flies still exhibit the classic increase in daytime sleep as temperature rises [4, 19, 30] (Figs. 2 and 3), indicating that mid-day sleep-promoting pathways are still functional despite its reduced impact. This suggests that *D. melanogaster* adapted to the cooler temperatures at high altitudes by modulating the thermal gradient controlling the propensity for sleep during the mid-day. Although it is not clear how a reduced mid-day siesta might enhance fitness in high altitude flies, it lengthens the timing of when flies can engage in daytime activities. Certainly, changes in altitude are also accompanied by other environmental changes besides temperature, such as a decrease in atmospheric pressure and increase in solar radiation. An increase in solar radiation with altitude might suggest the need to minimize daytime activity. However, recent evidence suggests that an increase in pigmentation at high altitudes is mainly directed at providing protection against elevated levels of UV irradiation [27]. Thus, adaption to high altitude likely involves adjusting to numerous environmental changes, with daytime temperature likely to be the key environmental cue influencing mid-day sleep behavior.

While not all the differences in sleep behavior as a function of altitude are shared by flies from different countries (e.g., Cameroon and Kenya), the results are reminiscent of those we observed comparing transgenic flies that either have the wildtype version of dmpi8 or a version where the 5’ and 3’ss were optimized leading to constitutively high splicing efficiency (termed M2M1 flies) [19]. For example, similar to what we observed when comparing low and high altitude flies, the reduced and more fragmented daytime sleep in M2M1 flies continues in constant light conditions (Fig. 4), indicating that altitudinal differences in daytime sleep behavior are not dependent on a functional circadian timing system. In addition, the average sleep bout length was substantially shorter in M2M1 flies, whereas there was less effect on sleep bout number. Likewise, high altitude flies had short sleep episodes compared to their low altitude counterparts but sleep bout number was less affected (Figs. 3 and 5). It is also interesting to note that the main effect of temperature on sleep levels is due to changes in sleep bout length as previously noted [19, 20], further suggesting that the adaptation of mid-day sleep behavior to high altitudes is mainly guided by thermal selection pressures. Thus, the relative difference in daytime sleep behavior between high and low altitude flies is somewhat analogous to what we observed for the M2M1 and control transgenic flies, respectively; i.e., both high altitude flies and M2M1 flies exhibit more of a cold phenotype in daily sleep/activity profiles characterized by reduced mid-day sleep, earlier onset of evening activity and a delayed offset in morning activity. The behavioral analogies between our earlier studies using transgenic flies with engineered differences in dmpi8 splicing efficiency and those based on using natural populations of *D. melanogaster* suggest that dmpi8 splicing contributed to the adaptation of flies to higher altitudes in tropical Africa.

Indeed, we show a strong correlation between dmpi8 splicing efficiency and altitudinal differences in sleep behavior at cooler temperatures that is intriguingly dependent on the SNP3 variant (Fig. 7e-j). Prior work established a causal relationship between increased dmpi8 splicing efficiency and reduced mid-day sleep, especially at cooler temperatures where the additional masking effects of heat and light are less potent [18]. Remarkably, at cooler temperatures, high altitude flies that carry the SNP3/A variant but not the SNP3/G variant showed higher daily levels of dmpi8 splicing compared to their low altitude counterparts, consistent with daytime sleep levels (Fig. 7e-j). The intrinsic differences in the dmpi8 splicing efficiencies of SNP3/A and SNP3/G flies offer an attractive explanation for why flies with SNP3/G exhibit less altitudinal variation in sleep behavior. Namely, the already more efficient splicing of dmpi8 in SNP3/G flies even at low altitudes creates a ‘ceiling’ effect on how much further dmpi8 splicing efficiency can be augmented at high altitudes. Indeed, there is less altitudinal difference in the splicing efficiency of dmpi8 splicing for flies with SNP3/G compared to SNP3/A (Fig. 7a-d). While the dmpi8 splicing results and daytime sleep levels as a function of SNP variant (Fig. 7a-d) and altitude (Fig. 7e-j) closely align at cooler temperatures, we note that a recent study indicated that a small subset of *per*-expressing clock cells in the brain are critical for mid-day siesta [35]. Therefore, we cannot rule out differential effects of dmpi8 splicing in some clock cells that are critical to mid-day sleep but not reflected in the whole head analysis we did.

Together, our findings suggest that dmpi8 splicing efficiency has played a greater role in the altitudinal differences in daytime sleep for natural population that carry the SNP3/A variant. Specifically, our findings suggest that evolutionary changes leading to boosting the levels of dmpi8 splicing efficiency above those normally found in lowland flies contributed to the adaptation of at least SNP3/A containing *D. melanogaster* from tropical Africa to the cooler temperatures at high altitudes. Based on our prior work showing that elevated levels of dmpi8 splicing lead to decreases in arousal thresholds [19], we propose that high altitude flies can more easily maintain wakefulness during the mid-day. Although dmpi8 splicing is more efficient in SNP3/G flies they still respond to warm temperatures by mounting a strong mid-day siesta (Fig. 2). As noted above, all dmpi8 introns that we examined to date have the identical suboptimal 5’ and 3’ss (data not shown). Since binding of the U1 snRNA to the 5’ss is the overall rate-limiting step in splicing [46], this likely ensures that dmpi8 splicing is strongly inhibited on warm days irrespective of any stimulatory effects from SNPs of other factors. In addition, mid-day activity is further suppressed by the ‘direct’ actions of heat and light. Thus, the elevated splicing of SNP3/G flies compared to SNP3/A flies is unlikely to be detrimental at warm temperatures. All these lines of reasoning suggest that the main influence of altering dmpi8 splicing efficiency would manifest itself in adapting to cooler temperatures where it has a stronger effect on setting mid-day sleep levels.

It is still not clear how changes in dmpi8 splicing efficiency affect mid-day siesta in a clock-independent manner. However, prior work has shown that increases in splicing efficiency lead to higher levels of *per* mRNA and protein [4, 17, 18]. Moreover, PER is expressed in brain cells that appear to have little effect on circadian rhythms but have prominent effects on mid-day sleep/arousal [35]. Thus, altitudinal changes in PER levels in key neurons could modulate mid-day sleep levels.

How dmpi8 splicing at cooler temperatures is increased in high altitude SNP3/A flies compared to their lowland counterparts is presently not known. We did not find any SNPs in the *per* 3’ UTR that show differences in frequency as a function of altitude (data not shown). It is possible that other adaptive changes at high altitudes, perhaps not even specific to dmpi8 splicing, lead to enhancement of dmpi8 intron removal. Nonetheless, there is still altitudinal variation in daytime sleep for flies with the SNP3/G variant (Fig. 6), which shows very little if any altitudinal changes in dmpi8 splicing efficiency (Fig. 7). These findings strongly suggest that for both SNP3/A and SNP3/G flies, other factors besides dmpi8 splicing also contributed to the altitudinal cline in mid-day sleep behavior. Future studies will be required to determine if SNP3/A or SNP3/G flies are ‘better’ adapted to high altitudes, although a lack of altitudinal difference in SNP3 frequency might suggest little difference and/or other compensatory adaptations. In this regard it is interesting to note that an earlier study using flies from the United States showed a latitudinal cline in mainly nighttime sleep with less effect on daytime sleep levels and no correlation with dmpi8 splicing efficiency [47]. It will be of interest to determine if this is due to a high frequency of SNP3/G containing flies in the populations studied from the United States. It is also possible that the more ancestral flies in Africa adapt differently to thermal clines compared to the more cosmopolitan strains in the United States. Irrespective, our findings indicate that comparative genomic analysis of high and low altitude flies from natural populations of *D. melanogaster* in tropical Africa should provide insights into the mechanisms regulating mid-day sleep and its thermal adaptation.

## Conclusions

In summary, we show that natural populations of *D. melanogaster* from tropical Africa living at high altitudes exhibit significantly reduced mid-day sleep levels and generally more fragmented sleep compared to their lowland counterparts. Since it is thought that the eastern lowlands of tropical Africa is the ancestral origins of *D. melanogaster*, it is understandable why this species has a genetically programmed mid-day sleep that exhibits thermal plasticity. However, higher altitudes are associated with cooler daytime temperatures, suggesting that reductions in mid-day siesta at high altitudes might be beneficial by lengthening the opportunity to engage in daytime behaviors when risk of heat exposure is minimized. Intriguingly, the altitudinal range in mid-day siesta is strongly regulated by a SNP in the *per* 3’ UTR that alters the intrinsic splicing efficiency of dmpi8. In both cases, though, the identical weak 5’ and 3’ss ensure that on warm days dmpi8 splicing is strongly inhibited, resulting in a robust mid-day siesta. Flies with the weaker splicing variant (SNP3/A) exhibit elevated dmpi8 splicing efficiency during cooler temperatures at high altitudes, suggesting that the ability to boost the splicing levels beyond those found in lowland flies contributed to the adaptation to the colder temperatures at higher elevations. In contrast, those with the SNP/G variant have higher splicing efficiency even at low altitudes, which apparently sets a ceiling that limits the ability to further increase dmpi8 splicing, thus attenuating the altitudinal differences in mid-day sleep for this group of flies. Nonetheless, both SNP3/A and SNP3/G flies show reduced mid-day siesta at high altitudes compared to their genotypic counterparts at low altitudes, suggesting that besides dmpi8 splicing, other factors contribute to the altitudinal cline in mid-day siesta. By comparing genetic differences between low and high altitude flies, which we are currently pursuing, this should lead to better insights into daytime sleep, which appear to be governed by mechanism distinct from those underlying nocturnal sleep.

## Methods

### Fly strains and general handling

All flies were routinely reared at room temperature (22-25 °C) and maintained in vials or bottles containing standard agar-cornmeal-sugar-yeast-Tegosept-media. All the *D. melanogaster* strains from natural populations in tropical Africa that we used in this study were descendants of flies generously obtained from the laboratories of Drs. Pool (University of Wisconsin), Begun (UC Davis) and Langley (UC Davis) for a total of 91 lines come from 20 localities in 10 countries (see Additional file 1: Table S1). Where known, references first describing published lines from a particular location are listed in Additional file 1: Table S1.

### Behavioral assays

Individual adult male flies (1–5 day-old) were placed in 65 mm × 5 mm glass tubes containing 5% sucrose with 2% Bacto agar. Locomotor activity was continuously monitored and recorded by using the Trikinetics (Waltham, MA, USA) system, as previously reported [16, 19]. Briefly, throughout the testing period flies were maintained at the indicated temperature (18°, 25° or 29 °C) and subjected to at least 5 days of 12 h light: 12 h dark cycles [LD; where zeitgeber time 0 (ZT0) is defined as lights-on]. Cool white fluorescent light (~1000 lux) was used during LD and the temperature did not vary by more than 0.5 °C between the light and dark periods. In general, after five days in LD, flies were kept in constant darkness (DD) or constant light (LL) for seven days. Data analysis of either locomotor activity or sleep parameters was done with the FaasX and Matlab programs, as previously described [16, 19]. Sleep was operationally defined as no detection of locomotor activity movement for any period of 5 contiguous min, which is routinely used in the field (e.g., [39, 40]). The values were based on pooling data from multiple individual flies of the same genotype. In general, for measuring sleep values or locomotor activity, the data for LD was an average of the last three LD days. For DD, the values were from single days. Free-running periods of locomotor activity rhythms was based on the data collected during six consecutive days in DD and using the Faasx program (kindly provide by F. Rouyer, France), as previously described [16, 19].

### Splicing assay

For RNA analysis in flies, vials containing ~40 adult flies were placed in controlled environmental chambers (Percival, USA) at the indicated temperatures and exposed to 5 days of LD. Every four hours at selected times during the last day in LD, flies were collected by freezing and heads isolated. Total RNA was extracted and the relative levels of dmpi8 spliced and unspliced *per* RNA variants in fly heads were measured using a semi-quantitative reserve transcriptase-PCR (RT-PCR) assay as previously described [18]. Briefly, RNA was collected from isolated fly heads using Tri-reagent (Sigma). Approximately 1 μg of total RNA was reverse transcribed using oligo dT and Thermoscript RT enzyme (Invitrogen or Clontech) in a 20 μl reaction. Gene specific primers flanking the 3’ UTR intron of dm*per* were used to amplify both the spliced and unspliced forms in a 25 μl reaction using 1 μl of RT product as template. The following primers were used to amplify the target regions: sense primer P6851f (5’ ACA CAG CAC GGG GAT GGG TAG T 3’) and antisense primer P7184r (5’ GGC TTG AGA TCT ACA TTA TCC TC 3’). The non-cycling Cap Binding Protein 20 (CBP20) gene was used as an internal control. The sense primer is CBP294f (5’ TGA TTG TGA TGG GCC TGG ACA AGT 3’), and the antisense primer is CBP536r (5’ GTC CAA GCG AGT GCC ATT CAC AAA 3’). PCR products were separated and visualized by electrophoresis on 2% agarose gels, and the bands were quantified using a Typhoon 9400 Imager.

## Additional files

Additional file 1: Table S1. List of African lines used in this study. (DOCX 26 kb)
Additional file 2: Figure S1. The effects of altitude on the daily wake-sleep profiles in females are similar to that observed in male flies. (a-h) Shown are group averages of the daily activity rhythms (a-d) and daily sleep levels (e-h) for adult female flies from low (red) and high (blue) altitudes representing several different locations in Cameroon (a, b, e, f) and Kenya (c, d, g, h). The fly lines used in this experiment were, CO1, CO16, CM16, CM54, KO6, KO10M, KM16 and KM20 (Additional file 1: Table S1). Flies were kept at 25 °C and entrained to five days of 12 h light/12 h dark cycles (LD) followed by constant dark conditions (DD). For each country, the locomotor activity data of individual flies (16 flies per line) from the same altitude group (low or high) were pooled. (a-d) To facilitate comparisons, the peak value in daily activity for each fly was set to 1.0 and the normalized profiles superimposed. For LD, the last three days’ worth of data was pooled; for DD, the first day is shown (DD1). Horizontal bars at bottom of panels denote 12-h periods of light (white bar), dark (black bar) and ‘subjective daytime’ in DD (gray bar). ZT, zeitgeber time (hr); CT, circadian time (hr). (PDF 32 kb)
Additional file 3: Table S2. Effect of altitude on period length in Cameroon and Kenya flies. (DOCX 15 kb)

## Abbreviations

CT: Circadian time (hr)
DD: Continuous dark
dmpi8: *Drosophila melanogaster period* gene intron 8
LD: 12 h/12 h light–dark cycle
LL: Continuous light
MSBL: Mean sleep bout length
ZT: Zeitgeber time (hr)
