# Supplementary material for: Mid-day siesta in natural populations of D. melanogaster from Africa exhibits an altitudinal cline and is regulated by splicing of a thermosensitive intron in the period clock gene
Source: BMC Evol Biol. 2017 Jan 23;17:32. doi: 10.1186/s12862-017-0880-8 (PMC5259850; doi:10.1186/s12862-017-0880-8)
Supplement: Additional file 1: Table S1. — List of African lines used in this study. (DOCX 26 kb) [file 12862_2017_880_MOESM1_ESM.docx]

**Table S1. List of African lines used in this study**

| Country | | Locality | Stock ID | Altitude (m) | Latitude | Longitude | SNP3 | Fly source*  (reference) | |
| --- | --- | --- | --- | --- | --- | --- | --- | --- | --- |
| Cameroon | | Oku | CO1 | 2169 | 6.25 | 10.43 | G | John Pool  (Pool et al., 2006, Genetics) | |
|  |  |  | CO2 | 2169 | 6.25 | 10.43 | G |  |  |
|  |  |  | CO4 | 2169 | 6.25 | 10.43 | A |  |  |
|  |  |  | CO8 | 2169 | 6.25 | 10.43 | A |  |  |
|  |  |  | CO10 | 2169 | 6.25 | 10.43 | G |  |  |
|  |  |  | CO13 | 2169 | 6.25 | 10.43 | G |  |  |
|  |  |  | CO15 | 2169 | 6.25 | 10.43 | G |  |  |
|  |  |  | CO16 | 2169 | 6.25 | 10.43 | A |  |  |
|  |  | Maroua | CM8 | 385 | 10.6 | 14.32 | G | John Pool  (Pool et al., 2006, Genetics) | |
|  |  |  | CM16 | 385 | 10.6 | 14.32 | A |  |  |
|  |  |  | CM17 | 385 | 10.6 | 14.32 | G |  |  |
|  |  |  | CM22 | 385 | 10.6 | 14.32 | G |  |  |
|  |  |  | CM54 | 385 | 10.6 | 14.32 | G |  |  |
|  |  | Nkouondja | CN3 | 1121 | 5.50 | 10.68 | G | John Pool  (Pool et al., 2006, Genetics) | |
|  |  | Yokadouma | CY1 | 561 | 3.52 | 15.05 | G | John Pool  (Pool et al., 2006, Genetics) | |
| Kenya | | Malindi | KM10 | 78 | -1.43 | 40.03 | A | John Pool  (Pool et al., 2006, Genetics) | |
|  |  |  | KM16 | 78 | -1.43 | 40.03 | A |  |  |
|  |  |  | KM20 | 78 | -1.43 | 40.03 | A |  |  |
|  |  |  | KM24 | 78 | -1.43 | 40.03 | A |  |  |
|  |  | Nyahururu | KN5 | 2303 | 0.035 | 36.36 | A | John Pool  (Pool et al., 2012, PLoS Genetics) | |
|  |  |  | KN6M | 2303 | 0.035 | 36.36 | A |  |  |
|  |  |  | KN11M | 2303 | 0.035 | 36.36 | A |  |  |
|  |  |  | KN13M | 2303 | 0.035 | 36.36 | G |  |  |
|  |  |  | KN19M | 2303 | 0.035 | 36.36 | A |  |  |
|  |  |  | KN23M | 2303 | 0.035 | 36.36 | G |  |  |
|  |  | Molo | KO2 | 2506 | -0.25 | 35.73 | A | John Pool  (Lack et al., 2015, Genetics) | |
|  |  |  | KO6 | 2506 | -0.25 | 35.73 | A |  |  |
|  |  |  | KO10M | 2506 | -0.25 | 35.73 | A |  |  |
|  |  | Marigat | KR55M | 1062 | 0.47 | 35.98 | G | John Pool  (Pool et al., 2012, PLoS Genetics) | |
|  |  |  | KR62M | 1062 | 0.47 | 35.98 | G |  |  |
|  |  |  | KR70M | 1062 | 0.47 | 35.98 | G |  |  |
| Ethiopia | | Dodola | ED1M | 2492 | 6.98 | 39.18 | A | John Pool  (Pool et al., 2012, PLoS Genetics) | |
|  |  |  | ED3M | 2492 | 6.98 | 39.18 | A |  |  |
|  |  |  | ED5M | 2492 | 6.98 | 39.18 | A |  |  |
|  |  |  | ED6M | 2492 | 6.98 | 39.18 | A |  |  |
|  |  | Ziway | EZ10 | 1642 | 7.93 | 38.72 | A | Charis Cardeno/Charles Langely  (Pool et al., 2012, PLoS Genetics) | |
|  |  |  | EZ34 | 1642 | 7.93 | 38.72 | A |  |  |
|  |  |  | EZ26S | 1642 | 7.93 | 38.72 | n.d. |  |  |
|  |  |  | EZ49S | 1642 | 7.93 | 38.72 | n.d. |  |  |
|  |  | Gambella | EA13N | 525 | 8.25 | 34.59 | n.d. | John Pool  (Bastide et al., 2014, BMC Evol. Biol.) | |
|  |  |  | EA22N | 525 | 8.25 | 34.59 | n.d. |  |  |
|  |  |  | EA59N | 525 | 8.25 | 34.59 | n.d. |  |  |
|  |  |  | EA84N | 525 | 8.25 | 34.59 | n.d. |  |  |
|  |  |  | EA87N | 525 | 8.25 | 34.59 | n.d. |  |  |
|  |  |  | EA90N | 525 | 8.25 | 34.59 | n.d. |  |  |
|  |  |  | EA126N | 525 | 8.25 | 34.59 | n.d. |  |  |
|  |  | Fiche | EF66N | 3070 | 9.81 | 38.63 | n.d. | John Pool  (Bastide et al., 2014, BMC Evol. Biol.) | |
|  |  |  | EF91N | 3070 | 9.81 | 38.63 | n.d. |  |  |
|  |  |  | EF93N | 3070 | 9.81 | 38.63 | n.d. |  |  |
|  |  |  | EF98N | 3070 | 9.81 | 38.63 | n.d. |  |  |
|  |  |  | EF108N | 3070 | 9.81 | 38.63 | n.d. |  |  |
|  |  |  | EF110N | 3070 | 9.81 | 38.63 | n.d. |  |  |
|  |  |  | EF120N | 3070 | 9.81 | 38.63 | n.d. |  |  |
| Uganda | | Namulonge | UG3 | 1134 | 0.53 | 32.60 | A | John Pool  (Pool et al., 2012, PLoS Genetics) | |
|  |  |  | UG5 | 1134 | 0.53 | 32.60 | A |  |  |
|  |  |  | UG6 | 1134 | 0.53 | 32.60 | A |  |  |
|  |  |  | UG11 | 1134 | 0.53 | 32.60 | G |  |  |
|  |  |  | UG17 | 1134 | 0.53 | 32.60 | A |  |  |
|  |  |  | UG28 | 1134 | 0.53 | 32.60 | A |  |  |
|  |  |  | UG54 | 1134 | 0.53 | 32.60 | A |  |  |
|  |  | Masindi | UM1 | 1170 | 1.68 | 31.72 | n.d. | Perot Saelao/David Begun  (Pool et al., 2012, PLoS Genetics) | |
|  |  |  | UM16 | 1170 | 1.68 | 31.72 | n.d. |  |  |
|  |  |  | UM102 | 1170 | 1.68 | 31.72 | n.d. |  |  |
| Tanzania | | Uyole | TZ1 | 1800 | -8.89 | 33.44 | n.d. | John Pool  (Pool et al., 2012, PLoS Genetics) | |
|  |  |  | TZ10 | 1800 | -8.89 | 33.44 | A |  |  |
|  |  |  | TZ14 | 1800 | -8.89 | 33.44 | A |  |  |
| Gabon | | Franceville | GA125 | 332 | -1.65 | 13.60 | n.d. | Perot Saelao/David Begun  (Pool et al., 2012, PLoS Genetics) | |
|  |  |  | GA129 | 332 | -1.65 | 13.60 | A |  |  |
|  |  |  | GA130 | 332 | -1.65 | 13.60 | A |  |  |
|  |  |  | GA145 | 332 | -1.65 | 13.60 | G |  |  |
|  |  |  | GA160 | 332 | -1.65 | 13.60 | G |  |  |
| Guinea | | Donde | GU3-3 | 801 | 10.70 | -12.25 | n.d. | Perot Saelao/David Begun  (Pool et al., 2012, PLoS Genetics) | |
|  |  |  | GU7 | 801 | 10.70 | -12.25 | A |  |  |
|  |  |  | GU10 | 801 | 10.70 | -12.25 | G |  |  |
|  |  |  | GU11-3 | 801 | 10.70 | -12.25 | n.d. |  |  |
| Nigeria | | Maiduguri | NG1N | 295 | 11.85 | 13.16 | G | Perot Saelao/David Begun  (Pool et al., 2012, PLoS Genetics) | |
|  |  |  | NG3N | 295 | 11.85 | 13.16 | G |  |  |
|  |  |  | NG6N | 295 | 11.85 | 13.16 | A |  |  |
|  |  |  | NG7 | 295 | 11.85 | 13.16 | G |  |  |
|  |  |  | NG9 | 295 | 11.85 | 13.16 | A |  |  |
| Rwanda | | Gikongoro | RG5 | 1927 | -2.49 | 28.92 | A | John Pool  (Pool et al., 2012, PLoS Genetics) | |
|  |  |  | RG7 | 1927 | -2.49 | 28.92 | G |  |  |
|  |  |  | RG10M | 1927 | -2.49 | 28.92 | A |  |  |
|  |  |  | RG15M | 1927 | -2.49 | 28.92 | A |  |  |
|  |  |  | RG18M | 1927 | -2.49 | 28.92 | G |  |  |
|  |  |  | RG34M | 1927 | -2.49 | 28.92 | A |  |  |
|  |  |  | RG36 | 1927 | -2.49 | 28.92 | A |  |  |
|  |  |  | RG39 | 1927 | -2.49 | 28.92 | A |  |  |
| Malawi | | Mwanza | MW6 | 618 | -15.62 | 34.52 | A | John Pool  (Pool et al., 2006, Genetics) | |
|  |  |  | MW11 | 618 | -15.62 | 34.52 | n.d. |  |  |
|  |  |  | MW28 | 618 | -15.62 | 34.52 | A |  |  |
| n.d., not determined  *, flies were obtained from the labs of Drs. John Pool (University of Wisconsin), David Begun (UC  Davis) and Charles Langely (UC Davis). References are given for published populations  in brackets. | | | | | | | |  | |
