# Supplementary figures and images for: Mid-day siesta in natural populations of D. melanogaster from Africa exhibits an altitudinal cline and is regulated by splicing of a thermosensitive intron in the period clock gene
Source: BMC Evol Biol. 2017 Jan 23;17:32. doi: 10.1186/s12862-017-0880-8 (PMC5259850; doi:10.1186/s12862-017-0880-8)

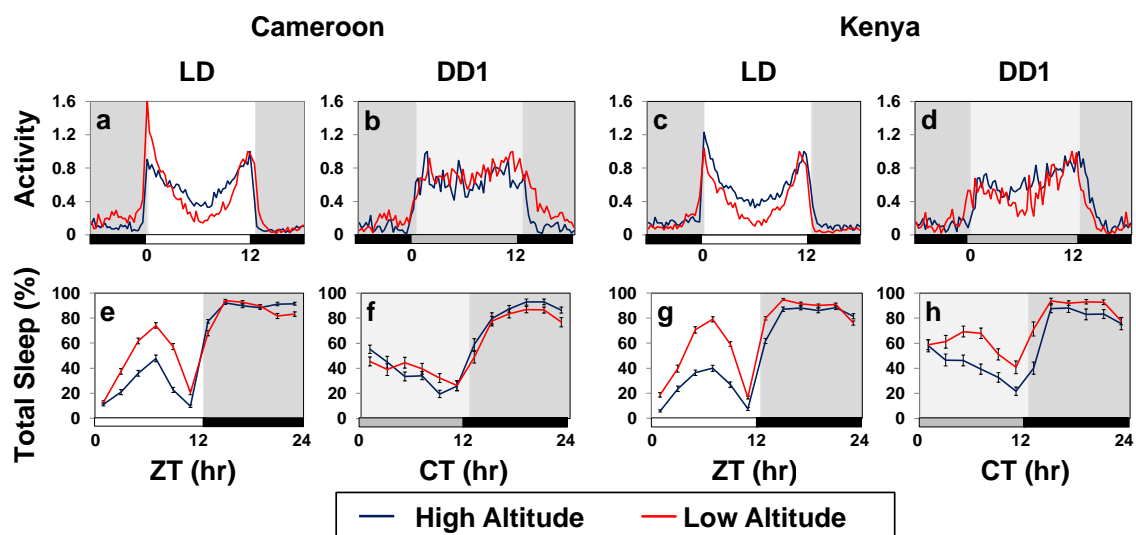

Supplement: Additional file 2: Figure S1. — The effects of altitude on the daily wake-sleep profiles in females are similar to that observed in male flies. (a-h) Shown are group averages of the daily activity rhythms (a-d) and daily sleep levels (e-h) for adult female flies from low (red) and high (blue) altitudes representing several different locations in Cameroon (a, b, e, f) and Kenya (c, d, g, h). The fly lines used in this experiment were, CO1, CO16, CM16, CM54, KO6, KO10M, KM16 and KM20 (Additional file 1: Table S1). Flies were kept at 25 °C and entrained to five days of 12 h light/12 h dark cycles (LD) followed by constant dark conditions (DD). For each country, the locomotor activity data of individual flies (16 flies per line) from the same altitude group (low or high) were pooled. (a-d) To facilitate comparisons, the peak value in daily activity for each fly was set to 1.0 and the normalized profiles superimposed. For LD, the last three days’ worth of data was pooled; for DD, the first day is shown (DD1). Horizontal bars at bottom of panels denote 12-h periods of light (white bar), dark (black bar) and ‘subjective daytime’ in DD (gray bar). ZT, zeitgeber time (hr); CT, circadian time (hr). (PDF 32 kb) [file 12862_2017_880_MOESM2_ESM.pdf]
