# Supplementary material for: Mid-day siesta in natural populations of D. melanogaster from Africa exhibits an altitudinal cline and is regulated by splicing of a thermosensitive intron in the period clock gene
Source: BMC Evol Biol. 2017 Jan 23;17:32. doi: 10.1186/s12862-017-0880-8 (PMC5259850; doi:10.1186/s12862-017-0880-8)
Supplement: Additional file 3: Table S2. — Effect of altitude on period length in Cameroon and Kenya flies. (DOCX 15 kb) [file 12862_2017_880_MOESM3_ESM.docx]

| **Table S2. Effect of altitude on period length in Cameroon and Kenya flies**   \| Temperature (°C) \| Country \| Altitude^a^ \| NumAlive^b^ \| %Rhythmic^c^ \| \| Period \| Power^d^ \| \| --- \| --- \| --- \| --- \| --- \| --- \| --- \| --- \| \| 18 \| Cameroon \| High \| 125 \| 91 \| 24.0 ± 0.1 \| \| 193.2 ± 6.6 \| \| Low \| 74 \| 97 \| 23.6 ± 0.1 \| \| 240.9 ± 7.8 \| \| Kenya \| High \| 137 \| 84 \| 23.9 ± 0.1 \| \| 205.0 ± 7.7 \| \| Low \| 32 \| 72 \| 23.5 ± 0.1 \| \| 160.8 ± 14.9 \| \| 25 \| Cameroon \| High \| 109 \| 95 \| 24.6 ± 0.1 \| \| 217.8 ± 6.3 \| \| Low \| 70 \| 99 \| 24.1 ± 0.1 \| \| 263.0 ± 8.9 \| \| Kenya \| High \| 129 \| 91 \| 24.7 ± 0.1 \| \| 208.5 ± 6.1 \| \| Low \| 43 \| 95 \| 23.9 ± 0.1 \| \| 182.8 ± 12.7 \| \| 29 \| Cameroon \| High \| 102 \| 90 \| 24.1 ± 0.1 \| \| 181.3 ± 6.0 \| \| Low \| 59 \| 93 \| 23.6 ± 0.0 \| \| 238.5 ± 9.2 \| \| Kenya \| High \| 127 \| 93 \| 24.1 ± 0.1 \| \| 209.7 ± 7.1 \| \| Low \| 34 \| 88 \| 23.9 ± 0.1 \| \| 203.3 ± 17.3 \| |
| --- | --- | --- | --- | --- | --- | --- | --- | --- | --- | --- | --- | --- | --- | --- | --- | --- | --- | --- | --- | --- | --- | --- | --- | --- | --- | --- | --- | --- | --- | --- | --- | --- | --- | --- | --- | --- | --- | --- | --- | --- | --- | --- | --- | --- | --- | --- | --- | --- | --- | --- | --- | --- | --- | --- | --- | --- | --- | --- | --- | --- | --- | --- | --- | --- | --- | --- | --- | --- | --- | --- | --- | --- | --- | --- | --- | --- | --- | --- | --- | --- | --- | --- | --- | --- | --- | --- | --- | --- | --- |

^a^ High altitude is 2169m~2506m, and low altitude is 78m~561m (see Table S1).

^b^ Total number of flies that survived throughout the entire testing period (5 days of LD and 7 days DD).

^c^ Flies with a power valve of greater than 10 and period ≥20 and ≤30, are defined as rhythmic.

^d^ Power is a measure of strength or amplitude of the rhythm.
